# (2*S*,4*S*)-3-Benzoyl-4-benzyl-2-*tert*-but­yl-1,3-oxazolidin-5-one

**DOI:** 10.1107/S1600536812035556

**Published:** 2012-08-23

**Authors:** Victoria J. Dungan, Helge Mueller-Bunz, Peter J. Rutledge

**Affiliations:** a School of Chemistry, The University of Sydney, NSW 2006, Australia; b School of Chemistry and Chemical Biology, University College Dublin, Belfield, Dublin 4, Ireland

## Abstract

In the title compound, C_21_H_23_NO_3_, the central oxazolidinone ring is approximately planar, the maximum deviation from the plane through the central ring being 0.043 (1) Å. The *tert*-butyl and benzyl substituents are *cis* to each other and *trans* to the *N*-benzoyl group. The inter­planar angle between the aromatic rings of the *C*-benzyl and *N*-benzoyl groups is 81.10 (4)°.

## Related literature

For background to this class of compound, see: Seebach & Naef (1981 ▶); Seebach *et al.* (1984 ▶); Seebach & Fadel (1985 ▶). For applications of these compounds in asymmetric synthesis, see: Krall *et al.* (2005 ▶); Barry & Rutledge (2008 ▶); Dungan *et al.* (2010 ▶, 2012 ▶). For related structures, see: Dungan *et al.* (2010 ▶); Barry *et al.* (2012 ▶).
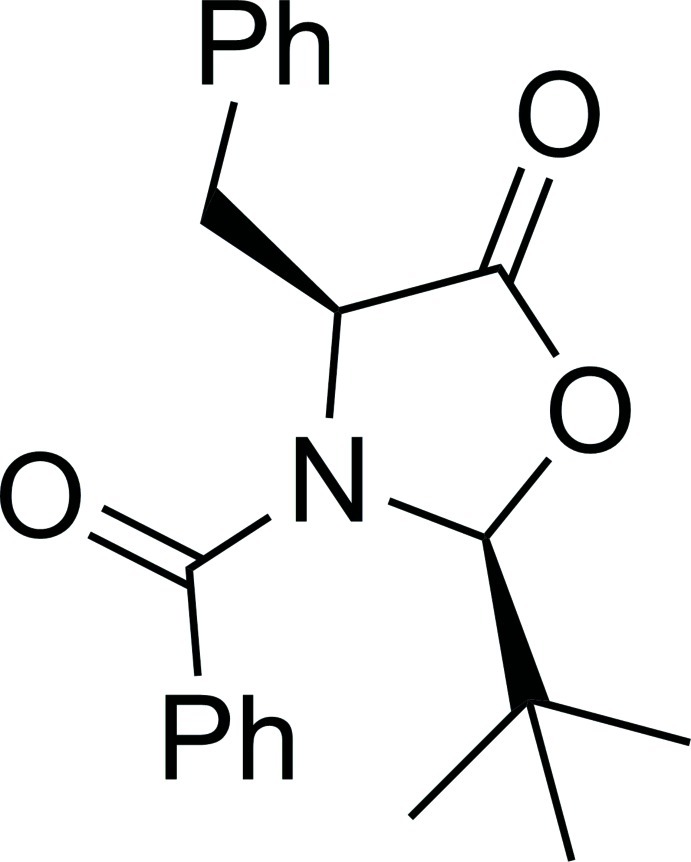


## Experimental

### 

#### Crystal data

C_21_H_23_NO_3_
*M* *_r_* = 337.40
Monoclinic, 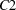

*a* = 23.6627 (17) Å
*b* = 7.1449 (5) Å
*c* = 12.2265 (9) Å
β = 117.470 (1)°
*V* = 1834.0 (2) Å^3^
*Z* = 4
Mo *K*α radiation
μ = 0.08 mm^−1^
*T* = 100 K
0.50 × 0.10 × 0.10 mm

#### Data collection

Bruker D8 platform diffractometer with SMART APEX CCD area detector
Absorption correction: multi-scan (*SADABS*; Sheldrick, 2000 ▶) *T*_min_ = 0.886, *T*_max_ = 0.992
15833 measured reflections
2390 independent reflections
2335 reflections with *I* > 2σ(*I*)
*R*_int_ = 0.019

#### Refinement

*R*[*F* ^2^ > 2σ(*F* ^2^)] = 0.034
*wR*(*F* ^2^) = 0.088
*S* = 1.05
2390 reflections
229 parameters
1 restraint
H-atom parameters constrained
Δρ_max_ = 0.32 e Å^−3^
Δρ_min_ = −0.19 e Å^−3^

### 

Data collection: *SMART* (Bruker, 2001 ▶); cell refinement: *SAINT* (Bruker, 2001 ▶); data reduction: *SAINT*; program(s) used to solve structure: *SHELXS97* (Sheldrick, 2008 ▶); program(s) used to refine structure: *SHELXL97* (Sheldrick, 2008 ▶); molecular graphics: *SHELXTL* (Sheldrick, 2008 ▶); software used to prepare material for publication: *SHELXL97*.

## Supplementary Material

Crystal structure: contains datablock(s) I, global. DOI: 10.1107/S1600536812035556/kj2203sup1.cif

Structure factors: contains datablock(s) I. DOI: 10.1107/S1600536812035556/kj2203Isup2.hkl

Supplementary material file. DOI: 10.1107/S1600536812035556/kj2203Isup3.cdx

Supplementary material file. DOI: 10.1107/S1600536812035556/kj2203Isup4.mol

Supplementary material file. DOI: 10.1107/S1600536812035556/kj2203Isup5.cml

Additional supplementary materials:  crystallographic information; 3D view; checkCIF report

**Fig. 1.**
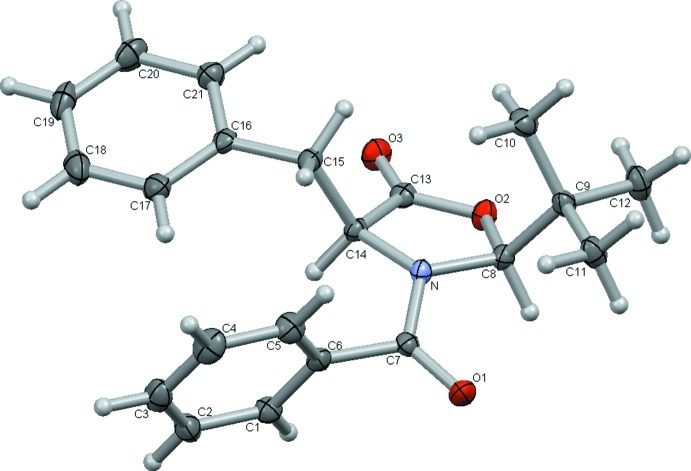
View of (2S,4S)-3-benzoyl-4-benzyl-2-(tert-butyl)oxazolidin-5-one showing displacement ellipsoids at the 50% probability level. Note the cis arrangement of the tert-butyl and benzyl groups.

Enhanced figure: interactive version of Fig. 1

## supplementary crystallographic information

### Comment

The stucture of the title compound (2*S*,4*S*)-3-benzoyl-4-benzyl-2-(*tert*-butyl)oxazolidin-5-one is shown below (Fig. 1). This structure reveals that the oxazolidinone ring is approximately planar, with the *tert*-butyl and benzyl groups occupying the same face of the ring plane. Observation of the *cis* isomer is in accord with previously reported NMR experiments (Seebach & Fadel, 1985) and the crystal structures of related compounds (Dungan *et al.* 2010; Barry *et al.* 2012). In the crystal, the angle between the oxazolidinone ring and the phenyl ring of the *N*-benzoyl group (C1 to C6) is 65.74 (4)°, while the angle between the oxazolidinone and the phenyl ring of the *C*-benzyl group (C16 to C21) is 25.66 (7)°.

Oxazolidinones of this type are of interest due to their capacity to undergo stereoselective α-alkylation *via* the cyclic enolate, a reaction that exploits the principle of "self-reproduction of chirality centres" introduced by Seebach and co-workers (Seebach & Naef, 1981, Seebach *et al.*, 1984, Seebach & Fadel, 1985). Thus the *tert*-butyl group directs an incoming electrophile to the opposite face of the planar enolate, giving an enantiopure product with retention of stereochemistry from the original oxazolidinone.

We have recently applied this strategy in the synthesis of ligand architectures designed to mimic the structure and function of non-heme iron enzymes (Krall *et al.* 2005, Barry & Rutledge, 2008, Dungan *et al.* 2010, Dungan *et al.* 2012, Barry *et al.* 2012)

### Experimental

The title compound was prepared following the procedure reported by Seebach (Seebach & Fadel, 1985). Thus the sodium salt of *L*-phenylalanine was condensed with pivalaldehyde, by heating at reflux in pentane overnight under Dean-Stark conditions to affect azeotropic removal of water. The intermediate Schiff base thus formed was treated with benzoyl chloride in dichloromethane at 233 K, prompting cyclization to give the crude product. Recrystallization from methanol gave white needles in a low overall yield (31%).

### Refinement

A floating origin restraint was automatically generated by *SHELXL*. Hydrogen atoms were added at calculated positions and refined using a riding model. Their isotropic displacement parameters were fixed to 1.2 (1.5 for methyl groups) times the equivalent one of the parent atom. C—H bond lengths range from 0.95 Å to 1.00 Å.

### Crystal data

**Table:**

C_21_H_23_NO_3_	*F*(000) = 720
*M**_r_* = 337.40	*D*_x_ = 1.222 Mg m^−^^3^
Monoclinic, *C*2	Mo *K*α radiation, λ = 0.71073 Å
Hall symbol: C 2y	Cell parameters from 9756 reflections
*a* = 23.6627 (17) Å	θ = 3.0–28.4°
*b* = 7.1449 (5) Å	µ = 0.08 mm^−^^1^
*c* = 12.2265 (9) Å	*T* = 100 K
β = 117.470 (1)°	Rod, colourless
*V* = 1834.0 (2) Å^3^	0.50 × 0.10 × 0.10 mm
*Z* = 4	

### Data collection

**Table:**

Bruker D8 platform diffractometer SMART APEX CCD area detector	2390 independent reflections
Radiation source: sealed tube	2335 reflections with *I* > 2σ(*I*)
Graphite monochromator	*R*_int_ = 0.019
Detector resolution: 8.366 pixels mm^-1^	θ_max_ = 28.5°, θ_min_ = 1.9°
φ and ω scans	*h* = −31→31
Absorption correction: multi-scan (*SADABS*; Sheldrick, 2000)	*k* = −9→9
*T*_min_ = 0.886, *T*_max_ = 0.992	*l* = −16→16
15833 measured reflections	

### Refinement

**Table:**

Refinement on *F*^2^	Primary atom site location: structure-invariant direct methods
Least-squares matrix: full	Secondary atom site location: difference Fourier map
*R*[*F*^2^ > 2σ(*F*^2^)] = 0.034	Hydrogen site location: inferred from neighbouring sites
*wR*(*F*^2^) = 0.088	H-atom parameters constrained
*S* = 1.05	*w* = 1/[σ^2^(*F*_o_^2^) + (0.0619*P*)^2^ + 0.4977*P*] where *P* = (*F*_o_^2^ + 2*F*_c_^2^)/3
2390 reflections	(Δ/σ)_max_ = 0.005
229 parameters	Δρ_max_ = 0.32 e Å^−^^3^
1 restraint	Δρ_min_ = −0.19 e Å^−^^3^

### Special details

**Table:**

Experimental. R(int) for selected reflections was 0.037 before and 0.019 after correction for absorption. The Ratio of minimum to maximum transmission is 0.893567. The λ/2 correction factor is 0.0015. Friedel pairs were merged.
Geometry. All e.s.d.'s (except the e.s.d. in the dihedral angle between two l.s. planes) are estimated using the full covariance matrix. The cell e.s.d.'s are taken into account individually in the estimation of e.s.d.'s in distances, angles and torsion angles; correlations between e.s.d.'s in cell parameters are only used when they are defined by crystal symmetry. An approximate (isotropic) treatment of cell e.s.d.'s is used for estimating e.s.d.'s involving l.s. planes.
Refinement. Refinement of *F*^2^ against ALL reflections. The weighted *R*-factor *wR* and goodness of fit *S* are based on *F*^2^, conventional *R*-factors *R* are based on *F*, with *F* set to zero for negative *F*^2^. The threshold expression of *F*^2^ > σ(*F*^2^) is used only for calculating *R*-factors(gt) *etc*. and is not relevant to the choice of reflections for refinement. *R*-factors based on *F*^2^ are statistically about twice as large as those based on *F*, and *R*- factors based on ALL data will be even larger.

### Fractional atomic coordinates and isotropic or equivalent isotropic displacement parameters (Å^2^)

**Table:**

	*x*	*y*	*z*	*U*_iso_*/*U*_eq_	
C1	0.32528 (7)	0.0666 (2)	0.72715 (13)	0.0201 (3)	
H1	0.2936	0.1615	0.6991	0.024*	
C2	0.35910 (8)	0.0255 (3)	0.85255 (14)	0.0238 (3)	
H2	0.3504	0.0929	0.9101	0.029*	
C3	0.40519 (8)	−0.1129 (3)	0.89347 (14)	0.0269 (4)	
H3	0.4285	−0.1390	0.9791	0.032*	
C4	0.41737 (8)	−0.2135 (3)	0.80964 (16)	0.0274 (3)	
H4	0.4485	−0.3100	0.8379	0.033*	
C5	0.38402 (7)	−0.1736 (2)	0.68398 (15)	0.0232 (3)	
H5	0.3924	−0.2422	0.6265	0.028*	
C6	0.33832 (7)	−0.0322 (2)	0.64354 (13)	0.0174 (3)	
C7	0.29922 (7)	0.0067 (2)	0.50761 (13)	0.0168 (3)	
O1	0.24806 (5)	−0.06990 (19)	0.44701 (10)	0.0243 (3)	
N	0.32549 (6)	0.12804 (19)	0.45687 (11)	0.0160 (3)	
C8	0.28720 (7)	0.1929 (2)	0.32915 (13)	0.0167 (3)	
H8	0.2410	0.1878	0.3077	0.020*	
C9	0.29772 (7)	0.0885 (2)	0.22984 (13)	0.0186 (3)	
C10	0.36825 (7)	0.0743 (3)	0.26437 (15)	0.0248 (3)	
H10A	0.3731	0.0156	0.1967	0.037*	
H10B	0.3901	−0.0016	0.3391	0.037*	
H10C	0.3870	0.2000	0.2795	0.037*	
C11	0.26947 (8)	−0.1078 (2)	0.21368 (14)	0.0240 (3)	
H11A	0.2922	−0.1782	0.2906	0.036*	
H11B	0.2738	−0.1725	0.1472	0.036*	
H11C	0.2243	−0.0989	0.1928	0.036*	
C12	0.26277 (8)	0.1979 (3)	0.10837 (14)	0.0269 (3)	
H12A	0.2636	0.1257	0.0410	0.040*	
H12B	0.2840	0.3185	0.1158	0.040*	
H12C	0.2185	0.2193	0.0910	0.040*	
O2	0.30612 (5)	0.38597 (16)	0.33203 (9)	0.0201 (2)	
C13	0.35521 (7)	0.4321 (2)	0.44092 (13)	0.0182 (3)	
O3	0.38072 (5)	0.58182 (17)	0.45787 (10)	0.0239 (2)	
C14	0.37239 (6)	0.2706 (2)	0.53073 (12)	0.0155 (3)	
H14	0.3649	0.3077	0.6019	0.019*	
C15	0.44249 (6)	0.2112 (2)	0.57882 (13)	0.0179 (3)	
H15A	0.4465	0.0764	0.5999	0.022*	
H15B	0.4547	0.2281	0.5121	0.022*	
C16	0.48839 (6)	0.3203 (2)	0.69091 (13)	0.0171 (3)	
C17	0.49970 (7)	0.2615 (3)	0.80782 (14)	0.0239 (3)	
H17	0.4764	0.1590	0.8162	0.029*	
C18	0.54481 (8)	0.3517 (3)	0.91234 (15)	0.0301 (4)	
H18	0.5519	0.3112	0.9917	0.036*	
C19	0.57940 (8)	0.4999 (3)	0.90157 (15)	0.0296 (4)	
H19	0.6111	0.5588	0.9733	0.035*	
C20	0.56771 (8)	0.5622 (3)	0.78598 (16)	0.0293 (4)	
H20	0.5910	0.6653	0.7782	0.035*	
C21	0.52189 (7)	0.4739 (2)	0.68114 (14)	0.0224 (3)	
H21	0.5134	0.5190	0.6019	0.027*	

### Atomic displacement parameters (Å^2^)

**Table:**

	*U*^11^	*U*^22^	*U*^33^	*U*^12^	*U*^13^	*U*^23^
C1	0.0200 (6)	0.0198 (7)	0.0207 (7)	0.0012 (6)	0.0096 (5)	0.0004 (6)
C2	0.0277 (8)	0.0263 (8)	0.0187 (7)	−0.0026 (6)	0.0117 (6)	−0.0014 (6)
C3	0.0270 (8)	0.0269 (8)	0.0213 (7)	−0.0038 (7)	0.0064 (6)	0.0055 (6)
C4	0.0262 (8)	0.0199 (8)	0.0313 (8)	0.0045 (6)	0.0093 (7)	0.0049 (7)
C5	0.0244 (7)	0.0191 (7)	0.0261 (7)	0.0007 (6)	0.0116 (6)	−0.0032 (6)
C6	0.0169 (6)	0.0178 (7)	0.0176 (6)	−0.0037 (6)	0.0079 (5)	−0.0013 (5)
C7	0.0169 (6)	0.0185 (7)	0.0164 (6)	−0.0011 (5)	0.0090 (5)	−0.0030 (5)
O1	0.0211 (5)	0.0310 (7)	0.0206 (5)	−0.0092 (5)	0.0095 (4)	−0.0036 (5)
N	0.0146 (5)	0.0178 (6)	0.0139 (5)	−0.0020 (5)	0.0051 (5)	−0.0030 (5)
C8	0.0157 (6)	0.0170 (7)	0.0154 (6)	−0.0014 (5)	0.0054 (5)	−0.0011 (5)
C9	0.0220 (7)	0.0187 (7)	0.0145 (6)	−0.0045 (6)	0.0079 (5)	−0.0022 (5)
C10	0.0254 (7)	0.0278 (8)	0.0247 (7)	−0.0024 (7)	0.0145 (6)	−0.0062 (7)
C11	0.0326 (8)	0.0205 (8)	0.0195 (6)	−0.0074 (7)	0.0124 (6)	−0.0042 (6)
C12	0.0348 (8)	0.0257 (8)	0.0169 (7)	−0.0033 (7)	0.0092 (6)	0.0023 (6)
O2	0.0205 (5)	0.0164 (5)	0.0197 (5)	−0.0009 (4)	0.0062 (4)	−0.0005 (4)
C13	0.0166 (6)	0.0181 (7)	0.0202 (6)	0.0016 (6)	0.0087 (5)	−0.0024 (6)
O3	0.0274 (6)	0.0173 (5)	0.0259 (5)	−0.0038 (5)	0.0113 (5)	−0.0034 (5)
C14	0.0139 (6)	0.0156 (6)	0.0165 (6)	−0.0024 (5)	0.0065 (5)	−0.0036 (5)
C15	0.0139 (6)	0.0188 (7)	0.0199 (6)	−0.0013 (6)	0.0068 (5)	−0.0036 (6)
C16	0.0126 (6)	0.0189 (7)	0.0188 (6)	0.0008 (5)	0.0065 (5)	−0.0024 (6)
C17	0.0189 (7)	0.0296 (8)	0.0223 (7)	0.0001 (6)	0.0087 (6)	0.0039 (7)
C18	0.0253 (7)	0.0419 (11)	0.0186 (7)	0.0069 (8)	0.0062 (6)	0.0013 (7)
C19	0.0207 (7)	0.0327 (9)	0.0250 (7)	0.0024 (7)	0.0017 (6)	−0.0119 (7)
C20	0.0247 (8)	0.0241 (8)	0.0350 (9)	−0.0071 (7)	0.0103 (7)	−0.0082 (8)
C21	0.0225 (7)	0.0222 (8)	0.0227 (7)	−0.0037 (6)	0.0105 (6)	−0.0022 (6)

### Geometric parameters (Å, º)

**Table:**

C1—C6	1.389 (2)	C11—H11A	0.9800
C1—C2	1.395 (2)	C11—H11B	0.9800
C1—H1	0.9500	C11—H11C	0.9800
C2—C3	1.384 (2)	C12—H12A	0.9800
C2—H2	0.9500	C12—H12B	0.9800
C3—C4	1.387 (3)	C12—H12C	0.9800
C3—H3	0.9500	O2—C13	1.3431 (17)
C4—C5	1.395 (2)	C13—O3	1.198 (2)
C4—H4	0.9500	C13—C14	1.514 (2)
C5—C6	1.393 (2)	C14—C15	1.5418 (18)
C5—H5	0.9500	C14—H14	1.0000
C6—C7	1.5090 (19)	C15—C16	1.5141 (19)
C7—O1	1.2194 (18)	C15—H15A	0.9900
C7—N	1.3706 (19)	C15—H15B	0.9900
N—C14	1.4695 (18)	C16—C21	1.391 (2)
N—C8	1.4728 (18)	C16—C17	1.392 (2)
C8—O2	1.4455 (19)	C17—C18	1.389 (2)
C8—C9	1.540 (2)	C17—H17	0.9500
C8—H8	1.0000	C18—C19	1.381 (3)
C9—C10	1.524 (2)	C18—H18	0.9500
C9—C11	1.527 (2)	C19—C20	1.383 (3)
C9—C12	1.539 (2)	C19—H19	0.9500
C10—H10A	0.9800	C20—C21	1.391 (2)
C10—H10B	0.9800	C20—H20	0.9500
C10—H10C	0.9800	C21—H21	0.9500
			
C6—C1—C2	119.49 (15)	H11A—C11—H11B	109.5
C6—C1—H1	120.3	C9—C11—H11C	109.5
C2—C1—H1	120.3	H11A—C11—H11C	109.5
C3—C2—C1	120.32 (15)	H11B—C11—H11C	109.5
C3—C2—H2	119.8	C9—C12—H12A	109.5
C1—C2—H2	119.8	C9—C12—H12B	109.5
C2—C3—C4	120.08 (14)	H12A—C12—H12B	109.5
C2—C3—H3	120.0	C9—C12—H12C	109.5
C4—C3—H3	120.0	H12A—C12—H12C	109.5
C3—C4—C5	120.20 (15)	H12B—C12—H12C	109.5
C3—C4—H4	119.9	C13—O2—C8	112.03 (12)
C5—C4—H4	119.9	O3—C13—O2	121.81 (14)
C6—C5—C4	119.44 (15)	O3—C13—C14	127.58 (14)
C6—C5—H5	120.3	O2—C13—C14	110.60 (13)
C4—C5—H5	120.3	N—C14—C13	102.04 (11)
C1—C6—C5	120.46 (14)	N—C14—C15	114.74 (12)
C1—C6—C7	119.02 (13)	C13—C14—C15	111.59 (12)
C5—C6—C7	120.39 (13)	N—C14—H14	109.4
O1—C7—N	122.64 (13)	C13—C14—H14	109.4
O1—C7—C6	121.26 (13)	C15—C14—H14	109.4
N—C7—C6	116.08 (12)	C16—C15—C14	113.58 (12)
C7—N—C14	122.12 (12)	C16—C15—H15A	108.9
C7—N—C8	119.56 (12)	C14—C15—H15A	108.9
C14—N—C8	110.75 (12)	C16—C15—H15B	108.9
O2—C8—N	104.03 (11)	C14—C15—H15B	108.9
O2—C8—C9	108.70 (12)	H15A—C15—H15B	107.7
N—C8—C9	116.05 (12)	C21—C16—C17	118.63 (14)
O2—C8—H8	109.3	C21—C16—C15	121.86 (13)
N—C8—H8	109.3	C17—C16—C15	119.45 (14)
C9—C8—H8	109.3	C18—C17—C16	120.44 (16)
C10—C9—C11	109.45 (14)	C18—C17—H17	119.8
C10—C9—C12	109.46 (13)	C16—C17—H17	119.8
C11—C9—C12	109.49 (12)	C19—C18—C17	120.37 (16)
C10—C9—C8	111.61 (12)	C19—C18—H18	119.8
C11—C9—C8	109.08 (12)	C17—C18—H18	119.8
C12—C9—C8	107.72 (13)	C18—C19—C20	119.76 (16)
C9—C10—H10A	109.5	C18—C19—H19	120.1
C9—C10—H10B	109.5	C20—C19—H19	120.1
H10A—C10—H10B	109.5	C19—C20—C21	119.95 (17)
C9—C10—H10C	109.5	C19—C20—H20	120.0
H10A—C10—H10C	109.5	C21—C20—H20	120.0
H10B—C10—H10C	109.5	C16—C21—C20	120.78 (15)
C9—C11—H11A	109.5	C16—C21—H21	119.6
C9—C11—H11B	109.5	C20—C21—H21	119.6
			
C6—C1—C2—C3	−0.1 (2)	N—C8—C9—C12	−170.29 (12)
C1—C2—C3—C4	−0.9 (3)	N—C8—O2—C13	7.13 (15)
C2—C3—C4—C5	1.1 (3)	C9—C8—O2—C13	−117.09 (13)
C3—C4—C5—C6	−0.2 (3)	C8—O2—C13—O3	174.27 (13)
C2—C1—C6—C5	1.1 (2)	C8—O2—C13—C14	−4.26 (16)
C2—C1—C6—C7	177.00 (14)	C7—N—C14—C13	−144.40 (13)
C4—C5—C6—C1	−0.9 (2)	C8—N—C14—C13	5.00 (14)
C4—C5—C6—C7	−176.77 (15)	C7—N—C14—C15	94.76 (16)
C1—C6—C7—O1	−84.5 (2)	C8—N—C14—C15	−115.84 (13)
C5—C6—C7—O1	91.41 (19)	O3—C13—C14—N	−178.96 (14)
C1—C6—C7—N	97.27 (16)	O2—C13—C14—N	−0.53 (15)
C5—C6—C7—N	−86.80 (18)	O3—C13—C14—C15	−56.0 (2)
O1—C7—N—C14	156.59 (15)	O2—C13—C14—C15	122.48 (13)
C6—C7—N—C14	−25.2 (2)	N—C14—C15—C16	−157.45 (12)
O1—C7—N—C8	9.8 (2)	C13—C14—C15—C16	87.12 (15)
C6—C7—N—C8	−172.04 (13)	C14—C15—C16—C21	−96.99 (16)
C7—N—C8—O2	142.88 (12)	C14—C15—C16—C17	85.79 (17)
C14—N—C8—O2	−7.41 (14)	C21—C16—C17—C18	−1.7 (2)
C7—N—C8—C9	−97.78 (16)	C15—C16—C17—C18	175.60 (15)
C14—N—C8—C9	111.93 (13)	C16—C17—C18—C19	−0.6 (3)
O2—C8—C9—C10	66.66 (16)	C17—C18—C19—C20	1.9 (3)
N—C8—C9—C10	−50.11 (18)	C18—C19—C20—C21	−0.9 (3)
O2—C8—C9—C11	−172.28 (12)	C17—C16—C21—C20	2.7 (2)
N—C8—C9—C11	70.95 (15)	C15—C16—C21—C20	−174.55 (15)
O2—C8—C9—C12	−53.52 (15)	C19—C20—C21—C16	−1.4 (3)
